# Curcumin protects human umbilical vein endothelial cells against high oxidized low density lipoprotein-induced lipotoxicity and modulates autophagy

**DOI:** 10.22038/IJBMS.2021.59969.13297

**Published:** 2021-12

**Authors:** Lifeng Zhao, Ruixi Luo, Honghong Yu, Shuaishuai Li, Qi Yu, Wenjia Wang, Kun Cai, Tao Xu, Rui Chen, Weiyi Tian

**Affiliations:** 1 Department of Immunology and Microbiology, School of Basic Medical Sciences, Guizhou University of Traditional Chinese Medicine, Guiyang 550025, P.R. China; 2 Department of Experimental Center, Guizhou University of Traditional Chinese Medicine, Guiyang 550025, P.R. China; 3 Department of Cardiovascular Medicine, Second Affiliated Hospital of Guizhou University of Traditional Chinese Medicine, Guiyang 550025, P.R. China; 4 Department of Morphology Laboratory, Guizhou University of Traditional Chinese Medicine, Guiyang 550025, P.R. China; # These authors contributed to the work equally and should be regarded as co-first authors

**Keywords:** Atherosclerosis, Autophagy, Curcumin, Endothelial cells, Lipid metabolism disorders

## Abstract

**Objective(s):**

Endothelial dysfunction is a precursor of cardiovascular disease, and protecting endothelial cells from damage is a treatment strategy for atherosclerosis (AS). Curcumin, a natural polyphenolic compound, has been shown to protect endothelial cells from dysfunction. In the present study, we investigated whether curcumin could ameliorate high oxidized low-density lipoprotein (ox-LDL)-induced endothelial lipotoxicity by inducing autophagy in human umbilical vein endothelial cells (HUVECs).

**Materials and Methods:**

HUVECs were treated with 50 μM high ox-LDL alone or in combination with 5 μM curcumin for 24 hr. Cell viability and function were assessed by the cell counting kit-8 (CCK-8) assay, tube formation assay and cell migration experiments. Oil red O staining was used to detect lipid droplet accumulation in HUVECs. The change in reactive oxygen species (ROS) levels in HUVECs was measured with the probe DCFH-DA. Quantitative real-time PCR (qPCR) and Western blotting were used to evaluate the mRNA and protein levels of several inflammatory and autophagy-related factors.

**Results:**

Cell viability was restored, tube formation and migration ability were increased, and lipid accumulation, oxidative stress and inflammatory responses were decreased in the curcumin-treated group compared with the high ox-LDL group. Furthermore, high ox-LDL inhibited HUVEC autophagy, and this effect was reversed by curcumin. Moreover, curcumin regulated the expression of several key proteins involved in the AMPK/mTOR/p70S6K signaling pathway.

**Conclusion:**

Our findings suggest that curcumin is able to reduce endothelial lipotoxicity and modulate autophagy and that the AMPK/mTOR/p70S6K pathway might play a key role in these effects.

## Introduction

The development of atherosclerosis (AS) leads to cardiovascular disease, which is the main cause of mortality worldwide. It has been reported that endothelial dysfunction is a precursor of AS (1). Multiple risk factors, such as hypertension (2), diabetes mellitus (3), and smoking (4), lead to endothelial activation and damage. Endothelial dysfunction occurs in vulnerable areas of the arterial vascular system, leading to the earliest detectable changes associated with the development of atherosclerotic lesions (5, 6), i.e., local infiltration, capture and physicochemical modification of circulating lipoprotein granules in the subendothelial space (7). This initiates a complex pathogenic sequence (8, 9). Furthermore, it has been reported that oxidized low-density lipoprotein (ox-LDL) can induce the expression of inflammatory factors, chemokines and adhesion molecules in endothelial cells, leading to endothelial dysfunction (10, 11). Many studies have indicated that ameliorating endothelial cell dysfunction induced by ox-LDL helps decrease the incidence of cardiovascular disease events (12, 13). Protecting the normal function of endothelial cells is a strategy for the treatment of vascular diseases.

Autophagy is a lysosome-mediated process by which intracellular components are self-digested and transported (14) and is induced by cellular stressors. Increasing evidence suggests that autophagy is involved in many physiological processes, including cell survival, cell metabolism, and host defense (15). In addition, autophagy is associated with cardiovascular diseases and is conducive to endothelial function (16, 17). Many investigators have reached a consensus that autophagy plays a significant role in protecting cells from oxidative stress (18), promoting cholesterol excretion (19), reducing apoptosis (20) and enhancing the stability of lesions under atherosclerotic conditions (21). These facts indicate that the induction of autophagy may be a potential therapeutic method for AS.

Autophagy is modulated by multiple signaling pathways, including the AMP-activated protein kinase (AMPK)/mammalian target of rapamycin (mTOR)/p70 ribosomal S6 protein kinase (p70S6K) pathway. AMPK is a major intracellular energy sensor and positive regulator of autophagy (22). AMPK has been reported to regulate autophagy by inhibiting mTOR and p70S6K signaling (23, 24). The mTOR and p70S6K pathways negatively regulate autophagy and play a significant role in the modulation of autophagy (25). Various studies have suggested that the regulation of vascular endothelial autophagy through the AMPK/mTOR/p70S6K signaling pathway can reduce the occurrence and prevent the development of disease (26, 27).

Curcumin is a natural polyphenolic compound found in the rhizome of the traditional Chinese medicine *Curcuma longa* and has multiple pharmacological activities, including anti-AS (28), anti-inflammatory (29), and antitumor (30) effects. It has been widely reported to have protective effects against AS. Zhang *et al*. (31) showed that curcumin can protect against AS in apoE knockout mice by suppressing the expression of TLR4. In addition, Zou *et al*. (32) showed that curcumin supplementation can suppress intestinal cholesterol absorption and prevent AS in ApoE knockout mice fed a high-fat diet. Curcumin is a naturally occurring autophagy regulator that has both activating and inhibitory (33) effects on autophagy mechanisms. However, the exact molecular mechanism of curcumin as an autophagy regulator in AS remains to be determined. Therefore, the molecular mechanism by which curcumin regulates autophagy to reduce endothelial cell lipotoxicity is worth exploring. The purpose of this research was to investigate whether curcumin can ameliorate high ox-LDL-induced endothelial cell lipotoxicity by modulating the AMPK/mTOR/p70S6K autophagy signaling pathway and subsequently alleviating endothelial dysfunction.

## Materials and Methods

***Cell culture and treatment***

Human umbilical vein endothelial cells (HUVECs) were obtained from Zhongqiao Xinzhou Co., Ltd. (Shanghai, China). Endothelial cells were cultured in endothelial cell medium (ECM; ScienCell Corporation, Billerica, USA) supplemented with 5% FBS (ScienCell Corporation) and 1% penicillin-streptomycin at 37°C in a humidified atmosphere of 5% CO_2_/95% air. The HUVECs were divided into the following experimental groups: the control group, which consisted of normally cultured HUVECs; the curcumin-treated control group, which comprised cells treated with 5 μM curcumin (Absin, Shanghai, China); the high ox-LDL group, which consisted of cells exposed to 50 μg/ml high ox-LDL (Yiyuan Co., Ltd, Guangzhou, China) for 24 hr; the high ox-LDL + curcumin group, which comprised cells treated with 5 μM curcumin and 50 μg/ml high ox-LDL for 24 hr; the chloroquine (CQ) group, which consisted of cells treated with 10 μM CQ (Sigma, Missouri, USA) 8 hr before analysis; and the compound C group, which comprised cells treated with 20 nmol/l compound C (S7306, Selleck, Huston, USA) for 24 hr.

***Cell viability***

After the cells reached 90% confluency, 8000 HUVECs per well were seeded in 96-well plates overnight. The HUVECs were stimulated with high ox-LDL (25, 50, 75, or 100 μg/ml) for 24 hr in the presence or absence of curcumin (2.5, 5, 10, 20 μM). After treatment, cell viability was measured with a cell counting kit-8 (CCK-8, Dojindo, Japan). 

***Tube formation assay***

In brief, after treatment, HUVECs were seeded in 96-well plates previously coated with Matrigel Basement Membrane Matrix (356234, Corning, New York, USA). Tube formation was then photographed using a computer-assisted microscope and quantified by measuring the length and number of capillary structures using ImageJ software (Media Cybernetics, LP).

***Cell migration assay***

After treatment, HUVECs were seeded in the upper chamber of a Transwell plate with a pore size of 8 μm (Corning, New York, USA) and cultured in serum-free ECM. Complete ECM containing 5% FBS was added to the lower chamber, and the cells were cultured for 12 hr. The chamber was removed, cleaned with PBS 3 times, fixed with methanol for 15 min. The nonmigrated cells in the inner layer of the chamber were wiped off with a cotton swab, and the cells in the outer layer of the chamber were stained with 0.1% crystal violet after being cleaned with PBS 3 times and incubated at room temperature for 15 min. The migrated cells were observed with a computer-assisted microscope. ImageJ software was used to analyze the number of migrated cells.

***Oil red O staining***

After treatment, HUVECs were fixed with 10% formalin for 10 min. Then, the cells were incubated with Oil red O (D027-1-1, NJJCBIO, Nanjing, China) for 30 min. Finally, the nuclei were labeled with hematoxylin. Images of HUVECs were captured using a computer-assisted microscope to assess lipid accumulation in HUVECs.

***ROS content measurement***

After treatment, HUVECs were washed with PBS, treated with the fluorescent probe DCFH-DA (300 μl; 10 mM) (Beyotime Co., Ltd, Shanghai, China), and incubated at 37 °C for 30 min. The mean fluorescence intensity was measured with a fluorescence microscope after staining.

***RNA isolation and quantitative real-time PCR (qPCR)***

TRIzol reagent (Ambion, Texas, USA) was used to isolate total RNA from cells, and 1.0 μg of RNA was reverse-transcribed into cDNA using a High-Capacity cDNA Synthesis Kit (Vazyme, Nanjing, China) following the manufacturer’s protocol. The expression of target genes was measured by real-time PCR, and the comparative threshold cycle (2^-^^△△^^CT^) method was used for analysis. The sequences of the primer used are listed in Table 1, and a commercial β-actin primer was obtained from Sangon Biotechnology (B661102, Shanghai, China). Each sample was tested in triplicate, and β-actin was used as an internal control.

***Western blot analysis***

RIPA lysis buffer (Solarbio, Beijing, China) and benzenesulfonyl fluoride were used to extract total protein. A BCA Protein Assay Kit (Epizyme Co., Ltd, Shanghai, China) was used to determine the protein concentration. Protein extracts were isolated, separated on a 12.5% SDS-PAGE gel and transferred onto a 0.22 μm PVDF membrane (Epizyme), which was incubated with the following primary antibodies: peroxisome proliferator-activated receptor γ (PPARγ) (16001-1-AP, Proteintech, Wuhan, China), interleukin-6 (IL-6) (ab233706, Abcam, Cambridge, 1:1000), interleukin-10 (IL-10) (ab133575, Abcam, 1:5000), tumor necrosis factor alpha (TNF-α) (17590-1-AP, Proteintech, Wuhan, China), microtubule-associated protein 1 light chain 3 (LC3) (2775S, Cell Signaling Technology, MA, USA, 1:1000), AMPK (HN0506, HuaBio, Hangzhou, China, 1:1000), mTOR (HN0824, HuaBio, 1:500), p70S6K (HJ0505, HuaBio, 1:1000), phosphorylated AMPK (p-AMPK) (bs-8813R, Bioss, Beijing, China, 1:1000), phosphorylated mTOR (p-mTOR) (2971S, Cell Signaling Technology, 1:500), and phosphorylated p70S6K (p-p70S6K) (9234S, Cell Signaling Technology, 1:1000). β-Actin (AC026, ABclonal, Wuhan, China, 1:50000) was used as an internal control. The membrane was then incubated with horseradish peroxidase (HRP)-conjugated secondary antibodies (BS13278, Bioworld Technology, Nanjing, China, 1:5000) for 1 hr at 37°C.

***Immunofluorescence***

Immunofluorescence was used to measure LC3 levels in HUVECs. Briefly, after treatment, HUVECs were treated with 4% buffered paraformaldehyde for 20 min. and then permeabilized with 0.1% Triton X-100 (Sigma) for 10 min. After being blocked with 1% BSA, the HUVECs were incubated with an anti-LC3-II antibody (1:200) overnight and then stained with secondary antibody (ab150077, Alexa Fluor 488-conjugated, Abcam, 1:200) for 1 h. Finally, the cells were incubated with DAPI (D9542, Sigma) for 5 min. The HUVECs were viewed and photographed using a fluorescence microscope (Olympus, Japan).

***Transmission electron microscopy (TEM)***

After HUVECs were fixed, dehydrated and embedded in Epon resin, they were stained with uranyl acetate and lead citrate solution and then analyzed by TEM.

***Statistical analyses***

All data are presented as the means ± SDs. Statistical analysis was performed by one-way ANOVA. If the results of one-way ANOVA were significant, Duncan’s multiple range test for multiple comparisons was performed. A value of *P* < 0.05 was considered statistically significant.

## Results

***Curcumin ameliorates high ox-LDL-induced lipotoxicity in HUVECs***

**Figure 1:**
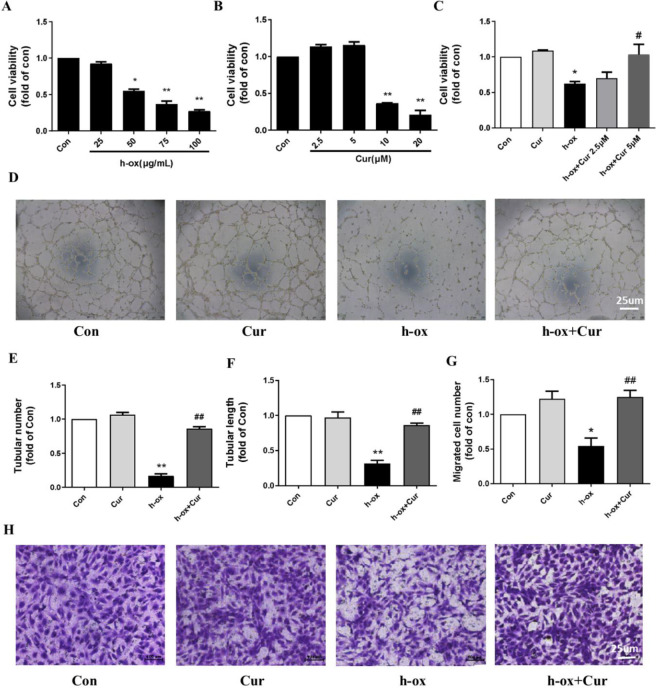
Effect of curcumin on the viability and function of HUVECs after high ox-LDL treatment (n=3). A: Dose-dependent effect of high ox-LDL (25 μg/ml, 50 μg/ml, 75 μg/ml, and 100 μg/ml) on HUVEC viability. B: Dose-dependent effects of curcumin (2.5 μM, 5 μM, 10 μM, and 20 μM) on HUVEC viability (n=3). C: HUVECs were exposed to high ox-LDL (50 μg/ml) in the presence or absence of curcumin (5 μM) for 24 hr, and cell viability was measured by the CCK-8 assay (n=3). D: Images of tubular structures formed by HUVECs. E and F: Tube number and tube length were quantified by using ImageJ software (n=3). G and H: Images and quantitative analysis of HUVEC migration in the Transwell experiment (n=3). The data are shown as the means±SDs (* *P*<0.05 vs. the control group; ** *P*<0.01 vs. the control group; # *P*<0.05 vs. the high ox-LDL group; ## *P*<0.01 vs. the high ox-LDL group). HUVECs, human umbilical vein endothelial cells; h-ox, highly oxidized low-density lipoprotein; Cur, curcumin

The CCK-8 assay suggested that high ox-LDL stimulation decreased the viability of HUVECs in a concentration-dependent manner (Figure 1A). After exposure to 50 μg/ml high ox-LDL, the cell viability decreased to 45%, and curcumin had no toxic effect on HUVECs at concentrations below 5 μM (Figure 1B). Then, we cultured HUVECs in the presence of 50 μM high ox-LDL alone or in combination with 2.5 μM or 5 μM curcumin. The results suggested that the viability of HUVECs was reduced by 40% in the group challenged with high ox-LDL for 24 hr compared with the control group and that 5 μM curcumin significantly restored cell viability. However, 2.5 μM curcumin only slightly restored cell viability (not significantly). Therefore, a curcumin concentration of 5 μM was selected for the rest of the experiments (Figure 1C). Tubule formation ability is an important indicator of HUVEC function in vitro. Under normal conditions, HUVECs can form a clear, honeycomb-shaped independent tubular structure on a substrate-coated surface. However, after high ox-LDL stimulation, the tube formation function of HUVECs was severely impaired, and the ability to form independent tubular structures was almost abolished. Curcumin significantly improved the tube formation ability of HUVECs (Figure 1D). Furthermore, the number and length of the formed tubules were significantly reduced after high ox-LDL stimulation and were restored after curcumin exposure (Figure 1E and 1F). Similarly, migration experiments indicated that the number of migrating cells was decreased after high ox-LDL stimulation and restored after curcumin exposure (Fig. 1G and 1H). In conclusion, curcumin alleviated high ox-LDL-induced lipotoxicity in HUVECs.

***Curcumin modulates lipid metabolism disorder in HUVECs induced by high ox-LDL***

**Figure 2:**
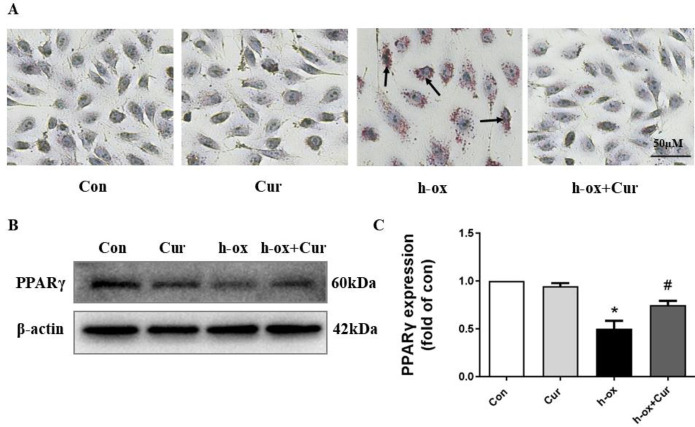
Effect of curcumin on lipid metabolism in HUVECs after high ox-LDL treatment. A: Oil Red O staining revealed lipid accumulation under a microscope (n=3). B and C: The protein expression levels of PPARγ were measured by Western blotting. PPARγ expression was quantified by densitometry using ImageJ software (n=3). The data are shown as the means±SDs (* *P*<0.05 vs. the control group; # *P*<0.05 vs. the high ox-LDL group). PPARγ, peroxisome proliferation-activated receptor γ

Lipid accumulation is a significant index of lipid metabolism. Therefore, we used Oil Red O staining to observe lipid droplets in cells. A large number of lipid droplets was observed in HUVECs in the high ox-LDL group, and curcumin treatment effectively reduced lipid accumulation after high ox-LDL treatment (Figure 2A). Moreover, the protein level of PPARγ was decreased after high ox-LDL stimulation and restored after curcumin exposure (Figure 2B). These results indicated that curcumin had the ability to modulate HUVEC lipid metabolism.

***Curcumin alleviates inflammation and oxidative stress induced by high ox-LDL in HUVECs***

**Figure 3:**
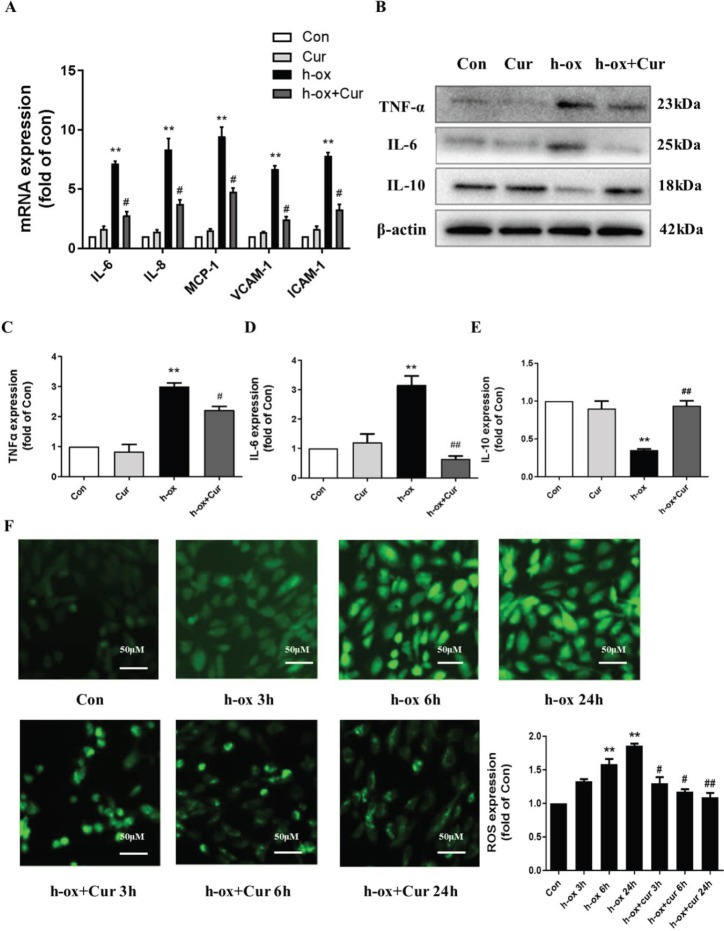
Effect of curcumin on inflammatory reactions and oxidative stress in HUVECs after high ox-LDL treatment. A: The mRNA expression levels of IL-6, IL-8, MCP-1, ICAM-1 and VCAM-1 were measured by quantitative PCR (n=3). B-E: The protein expression levels of TNFα, IL-6 and IL-10 were assessed by Western blotting. Expression was quantified by densitometry using ImageJ software (n=3). F: ROS production was evaluated by the fluorescent probe DCFH-DA (n=3). The data are shown as the means±SDs (** *P*<0.01 vs. the control group; # *P*<0.05 vs. the high ox-LDL group; ## *P*<0.01 vs. the high ox-LDL group). IL-6, interleukin-6; IL-8, interleukin-8; IL-10, interleukin-10; MCP-1, monocyte chemotactic protein-1; ICAM-1, intercellular cell adhesion molecule-1; VCAM-1, vascular cell adhesion molecule-1; TNFα, tumor necrosis factor alpha; ROS, reactive oxygen species

Under normal conditions, HUVECs express low levels of inflammatory factors. High ox-LDL obviously increased the expression of many inflammation-related genes (IL-6, interleukin-8 (IL-8), MCP-1, ICAM-1, and VCAM-1) and proteins (IL-6 and TNF-α), and curcumin markedly inhibited this change, indicating that it had powerful anti-inflammatory effects (Figure 3A-E). Moreover, high ox-LDL increased the level of reactive oxygen species (ROS) in HUVECs in a time-dependent manner, whereas the level of ROS decreased after curcumin treatment (Figure 3F). In general, these results suggested that curcumin ameliorated high ox-LDL-induced inflammation and oxidative stress in HUVECs.

***High ox-LDL inhibits autophagy in HUVECs***

**Figure 4:**
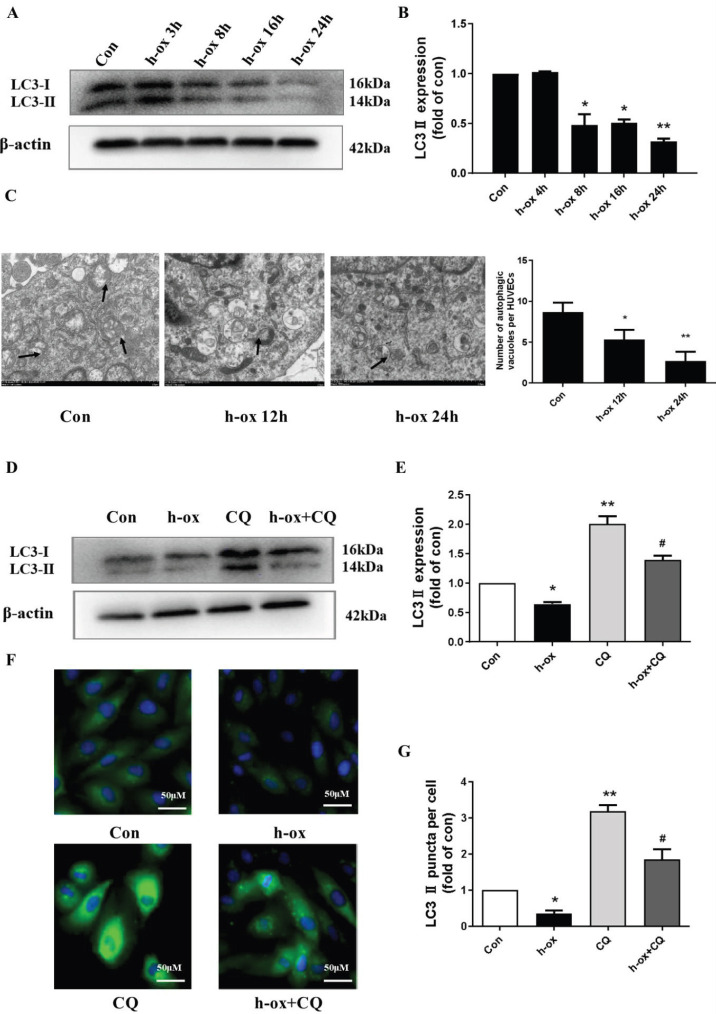
High ox-LDL inhibited autophagy in HUVECs. A and B: HUVECs were exposed to high ox-LDL (50 μg/mL) for the indicated times, and LC3-II protein levels were measured by Western blotting. Expression was quantified by densitometry using ImageJ software (n=3). C: HUVECs were exposed to high ox-LDL (50 μg/mL) for the indicated times, and autophagic vacuoles (black arrows) were detected by electron microscopy (n=3). D and E: HUVECs were treated with high ox-LDL for 16 h in the presence or absence of CQ (10 μM). CQ was added 8 h before the expression of LC3-II in the cell lysates was measured by Western blotting. Expression was quantified by densitometry using ImageJ software (n=3). F and G: Changes in LC3 immunofluorescence. Expression was quantified using ImageJ software (n=3). Autophagic vesicle accumulation was quantitated using ImageJ software and is expressed as the number of autophagic vesicles per cell. The data are shown as the means±SDs (* *P*<0.05 vs. the control group; # *P*< 0.05 vs. the high ox-LDL group; ## *P*<0.01 vs. the high ox-LDL group). LC3, microtubule-associated protein 1 light chain 3; CQ, chloroquine

Next, we evaluated whether high ox-LDL can affect autophagy in HUVECs. Western blot analysis showed that high ox-LDL reduced the protein level of the autophagy marker LC3-II in a time-dependent manner (Figure 4A and 4B). Electron microscopy also indicated that high ox-LDL inhibited the production of autophagic vacuoles in HUVECs (Figure 4C). Furthermore, we evaluated autophagic flux. HUVECs were treated with high ox-LDL in the presence or absence of CQ, a lysosomal inhibitor, which was used to block the degradation of autophagic vacuoles. Under normal conditions, CQ caused significant accumulation of the LC3-II protein, but stimulation of high ox-LDL diminished this effect (Figure 4D and 4E). Consistently, an increase in LC3 accumulation was not observed in the group treated with CQ and high ox-LDL compared with the group treated with CQ alone (Figure 4F and 4G), indicating that the number of autophagic vacuoles was decreased and that autophagy was blocked by high ox-LDL. In conclusion, these results showed that high ox-LDL inhibited autophagy in HUVECs.

***Curcumin ameliorates the high ox-ldl-induced inhibition of autophagy in HUVECs***

**Figure 5:**
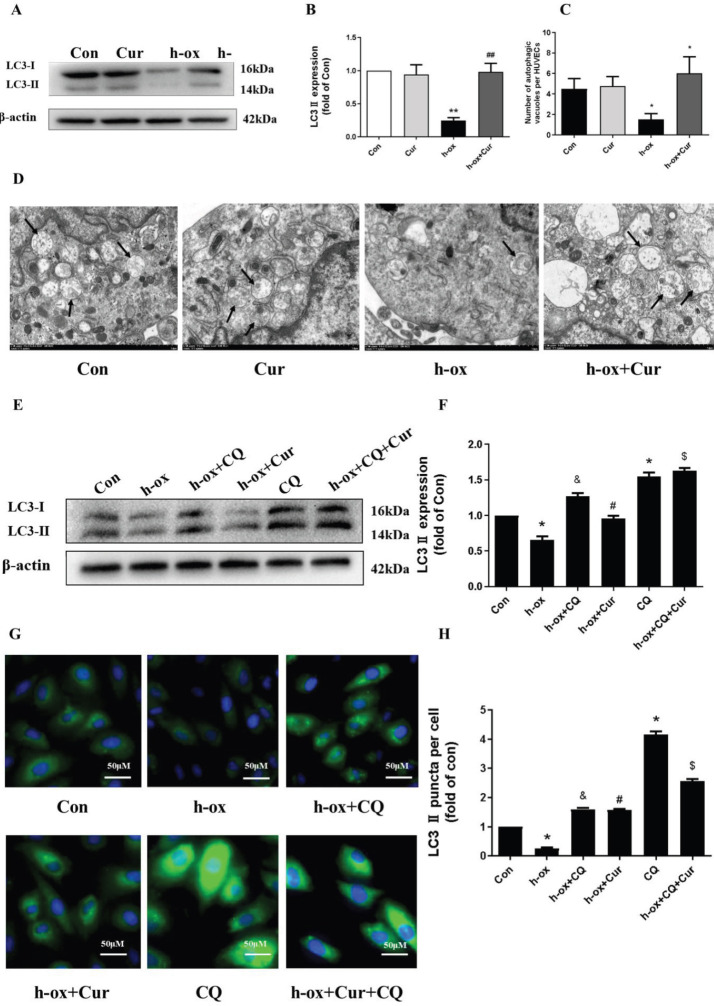
Curcumin ameliorated high ox-LDL-induced inhibition of autophagy in HUVECs. A and B: The protein expression levels of LC3-II were measured by Western blotting. LC3-II expression was quantified by densitometry using ImageJ software (n=3). C and D: Ultrastructural images of autophagic vacuoles (black arrows) in HUVECs (n=3). E and F: The protein expression levels of LC3-II were measured by Western blotting, and CQ was added 8 h before the assessment of autophagic flux. LC3-II expression was quantified by densitometry using ImageJ software (n=3). G and H: Immunofluorescence staining of LC3. Expression was quantified using ImageJ software (n=3). Autophagic vesicle accumulation was quantitated using ImageJ software and is expressed as the number of autophagic vesicles per cell. The data are shown as the means±SDs (* *P*< 0.05 vs. the control group; ** *P*<0.01 vs. the control group; # *P*<0.05 vs. the high ox-LDL group; ## *P*<0.05 vs. the high ox-LDL group; & *P*<0.05 vs. the CQ group; $ *P*<0.05 vs. the high ox-LDL +curcumin group)

We then evaluated whether curcumin can affect autophagy in HUVECs. The Western blot results showed that curcumin increased the protein expression of LC3-II, which was inhibited by high ox-LDL, while curcumin alone had no effect on the LC3-II level (Figure 5A and 5B). Similarly, under normal conditions, HUVECs have a typical paving stone shape with a clear cell outline, and electron microscopy showed that curcumin increased the production of autophagic vacuoles in HUVECs (Figure 5C and 5D). Furthermore, we evaluated autophagic flux by using CQ. Curcumin reversed the decrease in LC3-II protein levels induced by high ox-LDL, and CQ treatment enhanced this effect, suggesting that curcumin treatment restored autophagic flux (Figure 5E and 5F). The LC3 immunostaining results were similar (Figure 5G and 5H). These data collectively suggested that curcumin restored autophagy, which was inhibited by high ox-LDL.

***Curcumin ameliorates high ox-LDL-induced inhibition of autophagy through the AMPK/mTOR/p70S6K pathway***

**Figure 6:**
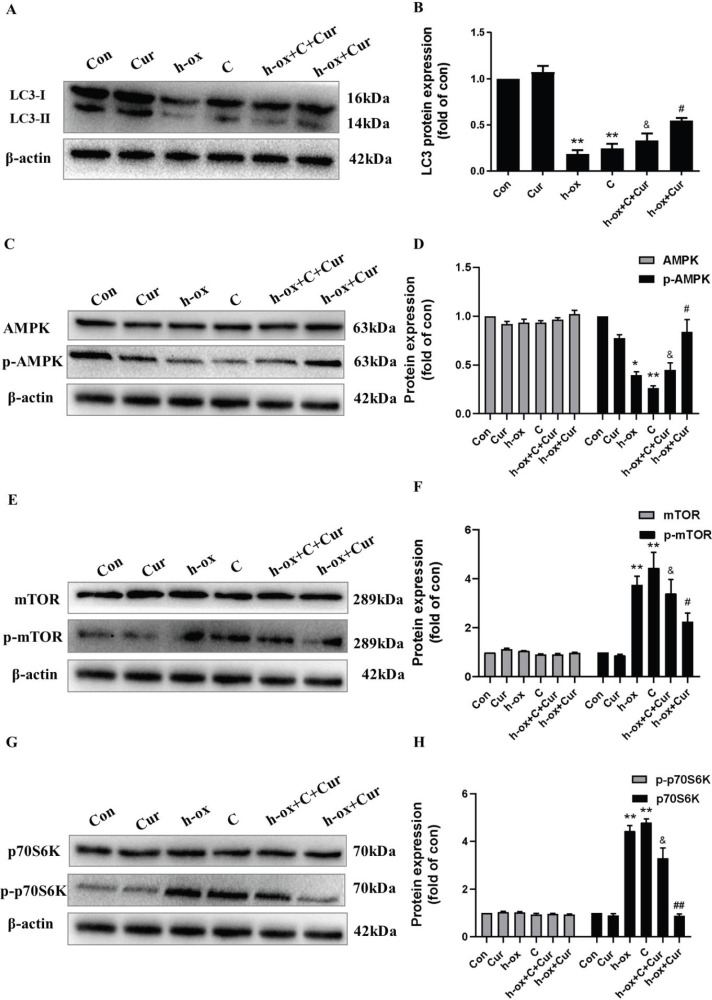
Curcumin ameliorated autophagy inhibition induced by high ox-LDL in HUVECs through the AMPK/mTOR/p70S6K pathway. A and B: The protein expression levels of LC3-II were measured by Western blotting (n=3). C and D: The protein expression levels of AMPK and p-AMPK were measured by Western blotting (n=3). E and F: The protein expression levels of mTOR and p-mTOR were measured by Western blotting (n=3). G and H: The protein expression levels of p70S6K and p-p70S6K were measured by Western blotting. Expression of these proteins was quantified by densitometry using ImageJ software (n=3). The data are shown as the means±SDs (* *P*< 0.01 vs. the control group; ** *P*< 0.01 vs. the control group; # *P*<0.05 vs. the high ox-LDL group; ##*P*< 0.01 vs. the high ox-LDL group; & *P*< 0.05 vs. the compound C group). AMPK, AMP-activated protein kinase; mTOR, mammalian target of rapamycin; p70S6K, p70 ribosomal S6 protein kinase

A large number of studies have suggested that the AMPK/mTOR/P70S6K pathway plays an important role in cardiovascular disease by strengthening autophagy and that suppression of the mTOR/P70S6K pathway through AMPK activation can enhance autophagic flux. Hence, we next evaluated whether curcumin can affect the AMPK/mTOR/p70S6K signaling pathway. The Western blot results indicated that high ox-LDL significantly suppressed the protein expression of p-AMPK and upregulated p-mTOR and p-p70S6K expression and that curcumin reversed these changes. To further verify our hypothesis, compound C, an AMPK inhibitor, was used in our study. Compound C significantly inhibited the protein expression of LC3 (Figure 6A and 6B), inhibited the protein expression of p-AMPK induced by curcumin (Figure 6C and 6D), and upregulated p-mTOR (Figure 6E and 6F) and p-p70S6K (Figure 6G and 6H) expression, demonstrating that AMPK/mTOR/p70S6K signaling is involved in the modulation of autophagy. These results proved that curcumin alleviated high ox-LDL-induced HUVEC damage by modulating the AMPK/mTOR/p70S6K pathway.

**Table 1:** Primers for real-time PCR

Gene	Forward	Reverse
IL-6	CCATTGCACAACTCTTTTCTCA	CTCCCAACAGACCTGTCTATAC
IL-8	CTGTTGGCCCAATTACTAACAG	TCCCGAATTGGAAAGGGAAATA
MCP-1	TCAAACTGAAGCTCGCACTCTCG	GGGAATGAAGGTGGCTGCTATGAG
ICAM-1	TGCAAGAAGATAGCCAACCAAT	GTACACGGTGAGGAAGGTTTTA
VCAM-1	CAGGCTGGAGATAGACTTACTG	CCTCAATGACAGGAGTAAAGGT

## Discussion

Here, we explored the curcumin-mediated amelioration of endothelial damage induced by high ox-LDL and the potential molecular mechanisms. Our findings demonstrated that high ox-LDL resulted in lipotoxicity and metabolic disturbance in HUVECs but that administration of curcumin alleviated high ox-LDL-induced cellular injury, including the impairment of tube formation and migration ability and increases in inflammation and oxidative stress, and thus improved endothelial function. Mechanistically, we found that curcumin plays a critical role in alleviating endothelial cell injury by regulating the autophagy-related AMPK/mTOR/p70S6K signaling pathway.

Normal endothelial cell function is essential for angiogenesis and a critical factor in tissue repair (34). Repairing injured endothelial cells is beneficial for the treatment of various vascular diseases. Yamagata showed (35) that docosahexaenoic acid (DHA) can reduce cardiovascular risk by controlling the production of nitric oxide and endothelin 1 in endothelial cells, affecting vasodilation and constriction. Wang *et al*. (36) indicated that interleukin-24 (IL-24) has a protective effect against H_2_O_2_-induced endothelial cell injury by reducing the levels of ROS and may thus be a new target for the treatment of cardiovascular disease. Curcumin was reported to have protective effects on impaired endothelial cells. Guo *et al*. (37) showed that curcumin antagonizes the detrimental effect of rapamycin on aortic endothelial cells in vitro by upregulating eNOS expression. Li *et al*. (38) suggested that curcumin is able to protect endothelial cells against homocysteine (HCY)-induced injury by suppressing NF-κB activation and downregulating IL-8 expression. Our previous work suggested that curcumin ameliorates palmitic acid (PA)-induced endothelial lipotoxicity and LOX-1 expression upregulation by reducing ER stress in HUVECs (39), prompting us to explore whether curcumin is able to alleviate high ox-LDL-induced endothelial injury. Finally, we found that curcumin reversed the damage to HUVECs induced by high ox-LDL, promoted tube formation and cell migration, and ameliorated inflammation and oxidative stress.

Triglyceride accumulation is an important manifestation of endothelial lipotoxicity induced by ox-LDL. Recent studies have reported that curcumin has regulatory effects on lipid accumulation by increasing FOXO3a activity (40) and lipoprotein metabolism (41). Similarly, in our study, curcumin reduced lipid accumulation in HUVECs induced by high ox-LDL. Additionally, the protein expression of PPARγ, a significant regulator of lipid metabolism, was increased after curcumin was added. Therefore, curcumin can modulate lipid metabolism disorder in HUVECs induced by high ox-LDL, but the detailed molecular mechanism remains to be explored.

Autophagy is a metabolic process necessary for mammalian homeostasis (42), especially for balancing energy homeostasis. Many studies have indicated that modulating autophagy might be beneficial for the treatment of cardiovascular disease (43). Liao *et al*. (44) showed that blocking autophagy makes macrophages more prone to cell death, aggravates the recognition and clearance of dead cells by exportin, and promotes plaque necrosis in ApoE^−/−^ mice with advanced AS. Razani *et al*. (45) provided evidence that autophagy deficiency leads to inflammasome hyperactivation, which further promotes AS progression. Moreover, activating autophagy may directly alleviate lipotoxicity. Sung-E Choi* et al*. found that autophagy agonists (AKTI and rapamycin) significantly increase autophagosome formation and prevent the PA-induced reduction in INS-1 cell viability (46). Furthermore, autophagy is now recognized as a process that has multiple functions in addition to balancing energy homeostasis. Cells also rely on autophagy to protect themselves against various harmful stimuli, such as oxidative stress and ER stress (47). Han *et al*. (48) indicated that curcumin has the potential for use as an autophagy-related antioxidant for the prevention and treatment of oxidative stress. Wang *et al*. (49) showed that vascular smooth muscle cells transform from the contraction phenotype to the synthetic phenotype when stimulated by ox-LDL and that autophagy is inhibited during this process. However, curcumin-mediated photodynamic therapy significantly increases the level of autophagy and inhibits the phenotypic transformation induced by ox-LDL. The above studies suggest that modulating autophagy might be critical for the curcumin-mediated protection of HUVECs. In this study, to determine the impact of curcumin on autophagy, we assessed the expression of LC3 and the production of autophagic vesicles and found that high ox-LDL inhibited autophagy in HUVECs. Interestingly, curcumin reversed the inhibitory effect of high ox-LDL on autophagy. To accurately assess the effect of high ox-LDL on autophagic flux, the lysosomal inhibitor CQ was added to inhibit the fusion and degradation of autophagosomes and lysosomes. Moreover, the effect of curcumin was further enhanced by CQ treatment, indicating that curcumin alleviated the inhibitory effect of high ox-LDL on autophagy in HUVECs. Taken together, our results suggested that autophagy modulation might play an important role in the protective effect of curcumin against lipotoxicity in HUVECs.

Although various pathways or signaling events may affect autophagy, the two major regulators of autophagy in mammalian cells are AMPK and mTOR kinases (50). AMPK is an important energy sensor that promotes autophagy by regulating cell metabolism to maintain energy homeostasis (51). Although the process by which autophagy is regulated is complex, with multiple signal cascades and modulatory mechanisms regulating autophagy activity, AMPK may be the most evolutionarily conserved autophagy inducer, and its activity is related to the autophagic degradation of almost all eukaryotic cells (52). Chen *et al*. (53) showed that melatonin activates autophagy through the AMPK/mTOR/ulk1 pathway, thus protecting VSMCs from calcification. In addition, AMPK can inhibit cardiac hypertrophy by stimulating autophagy through mTORC1 signaling (54). Guo *et al*. (55) indicated that curcumin may protect cells against oxidative stressinduced damage by inhibiting apoptosis and inducing autophagy via the Akt/mTOR pathway. mTOR is a downstream factor of AMPK, and AMPK activation can inhibit mTOR and block the phosphorylation of mTOR. p70S6K is a key downstream kinase of mTOR, and inhibition of mTOR prevents the phosphorylation of p70S6K. Therefore, follow-up experiments were conducted to determine whether curcumin induces autophagy through the AMPK/mTOR/p70S6K signaling pathway. The results showed that curcumin upregulated the expression of p-AMPK and downregulated the expression of p-mTOR and p-p70S6K, suggesting that curcumin activates AMPK and inhibits mTOR and p70S6K. Then, AMPK activity was blocked using compound C, an AMPK inhibitor, which inhibited curcumin-induced autophagy, indicating that curcumin activates autophagy through the AMPK/mTOR/p70S6K signaling pathway. AMPK is the main regulator of cellular lipid metabolism; once activated, AMPK can decrease the activity of enzymes that are crucial for lipid synthesis and related processes, suggesting that its activity is associated with the cellular energy level. However, whether the influence of curcumin on lipid metabolism, inflammation and oxidative stress is associated with the AMPK/mTOR/p70S6K pathway remains to be further explored. Moreover, whether the protective effect of curcumin on autophagy in HUVECs involves other pathways, such as chaperone-mediated autophagy or mitophagy, remains to be studied, and the in vivo effects of curcumin also need to be further investigated in depth in the future.

## Conclusion

Our results demonstrate that curcumin treatment attenuates high ox-LDL-induced endothelial damage and that curcumin-mediated modulation of autophagy via the AMPK/mTOR/p70S6K pathway might play a critical role in this effect. This study provides a new molecular mechanism by which curcumin protects against endothelial dysfunction.

## Authors’ Contributions

ZLF, LRX, and TWY conceived the study; ZLF and L RX performed the experiments and data collection with help from YHH, LSS, YQ, WWJ, CK, XT, and CR. ZLF and L RX wrote and TWY edited the paper.  

## Supplementary Material

All of the primers used are listed in the supplementary material (Supplementary Table S1).

## Conflicts of Interest

The authors declare no potential conflicts of interest with respect to the research, authorship, and/or publication of this article.

## References

[1] Endemann DH, Schiffrin EL (2004). Endothelial dysfunction. J Am Soc Nephrol.

[2] Bernhard D, Wang XL (2007). Smoking, oxidative stress and cardiovascular diseases-do anti-oxidative therapies fail?. Curr Med Chem.

[3] Nicolls MR, Haskins K, Flores SC (2007). Oxidant stress, immune dysregulation, and vascular function in type I diabetes. Antioxid Redox Signal.

[4] Buday A, Orsy P, Godó M, Mózes M, Kökény G, Lacza Z (2010). Elevated systemic TGF-beta impairs aortic vasomotor function through activation of NADPH oxidase-driven superoxide production and leads to hypertension, myocardial remodeling, and increased plaque formation in apoE(-/-) mice. Am J Physiol Heart Circ Physiol.

[5] Stary HC (2000). Natural history and histological classification of atherosclerotic lesions:an update. Arterioscler Thromb Vasc Biol.

[6] Virmani R, Kolodgie FD, Burke AP, Farb A, Schwartz SM (2000). Lessons from sudden coronary death: a comprehensive morphological classification scheme for atherosclerotic lesions. Arterioscler Thromb Vasc Biol.

[7] Simionescu N, Vasile E, Lupu F, Popescu G, Simionescu M (1986). Prelesional events in atherogenesis Accumulation of extracellular cholesterol-rich liposomes in the arterial intima and cardiac valves of the hyperlipidemic rabbit. Am J Pathol.

[8] Ross R, Glomset JA (1973). Atherosclerosis and the arterial smooth muscle cell: Proliferation of smooth muscle is a key event in the genesis of the lesions of atherosclerosis. Science.

[9] Ross R, Glomset JA (1976). The pathogenesis of atherosclerosis (first of two parts). N Engl J Med.

[10] Zhang X, Han X, Zhang P, Zhou T, Chen Y, Jin J (2019). Morin attenuates oxidized low-density lipoprotein-mediated injury by inducing autophagy via activating AMPK signalling in HUVECs. Clin Exp Pharmacol Physiol.

[11] Luo RX, Li LZ, Liu XH, Yuan YJ, Zhu WZ, Li L (2010). Mesenchymal stem cells alleviate palmitic acid-induced endothelial-to-mesenchymal transition by suppressing endoplasmic reticulum stress. Am J Physiol Endocrinol Metab.

[12] Yamagata K, Yamori Y (2020). Inhibition of endothelial dysfunction by dietary flavonoids and preventive effects against cardiovascular disease. J Cardiovasc Pharmacol.

[13] Tomonori A, Daisuke S, Noriaki T, Seiji T, Eiichiro Y, Yasuhiro I (2017). Effects of the mean amplitude of glycemic excursions and vascular endothelial dysfunction on cardiovascular events in nondiabetic patients with coronary artery disease. J Am Heart Assoc.

[14] Yang Z, Klionsky DJ (2010). Eaten alive: a history of macroautophagy. Nat Cell Biol.

[15] Puleston DJ, Simon AK (2014). Autophagy in the immune system. Immunology.

[16] Ouimet M (2013). Autophagy in obesity and atherosclerosis: interrelationships between cholesterol homeostasis, lipoprotein metabolism and autophagy in macrophages and other systems. Biochim Biophys Acta.

[17] Muller C, Salvayre R, Negre-Salvayre A, Vindis C (2011). Oxidized LDLs trigger endoplasmic reticulum stress and autophagy: prevention by HDLs. Autophagy.

[18] Perrotta I, Aquila S (2015). The role of oxidative stress and autophagy in atherosclerosis. Oxid Med Cell Longev.

[19] Le Guezennec X, Brichkina A, Huang YF, Kostromina E, Han W, Bulavin DV (2012). Wip1-dependent regulation of autophagy, obesity, and atherosclerosis. Cell Metab.

[20] Shan R, Liu N, Yan Y, Liu B (2020). Apoptosis, autophagy and atherosclerosis: relationships and the role of Hsp27. Pharmacol Res.

[21] Peng S, Xu LW, Che XY, Xiao QQ, Pu J, Shao Q (2018). Atorvastatin inhibits inflammatory response, attenuates lipid deposition, and improves the stability of vulnerable atherosclerotic plaques by modulating autophagy. Front Pharmacol.

[22] Zhang N, Zhi XY, Zhao J, Wei JL, Li JP, Yang HF (2020). Mesoporous silica induces hippocampal neurons cell autophagy through AMPK/mTOR/P70S6K signaling pathway. Environ Toxicol.

[23] Kim J, Kundu M, Viollet B, Guan KL (2011). AMPK and mTOR regulate autophagy through direct phosphorylation of Ulk1. Nat Cell Biol.

[24] Zhang P, Liu X, Li H, Chen Z, Yao X, Jin J (2017). TRPC5-induced autophagy promotes drug resistance in breast carcinoma via CaMKKbeta/AMPKalpha/mTOR pathway. Sci Rep.

[25] Kim Y C, Guan K L (2015). mTOR: a pharmacologic target for autophagy regulation. J Clin Invest.

[26] Sun XW, Wang DY, Zhang TT, Lu XJ, Duan FF, Ju LI (2020). Eugenol attenuates cerebral ischemia-reperfusion injury by enhancing autophagy via AMPK-mTOR-P70S6K pathway. Front Pharmacol.

[27] Wang P, Jiang LZ, Zhou N, Zhou H, Liu HZ, Zhao WR (2018). Resveratrol ameliorates autophagic flux to promote functional recovery in rats after spinal cord injury. Oncotarget.

[28] Mohammadian HS, Karimzadeh MR, Azhdari S, Vahedi P, Abdollahi E, Momtazi-Borojeni AA (2020). Modulatory effects of curcumin on the atherogenic activities of inflammatory monocytes: Evidence from in vitro and animal models of human atherosclerosis. Biofactors.

[29] Zhou YY, Zhang TT, Wang XF, Wei XW, Chen YZ, Guo LY (2015). Curcumin modulates macrophage polarization through the inhibition of the Toll-like receptor 4 expression and its signaling pathways. Cell Physiol Biochem.

[30] Batra H, Pawar S, Bahl D (2019). Curcumin in combination with anti-cancer drugs: A nanomedicine review. Pharmacol Res.

[31] Zhang S, Zou J, Li P, Zheng X, Feng D (2018). Curcumin protects against atherosclerosis in apolipoprotein E-Knockout mice by inhibiting Toll-like receptor 4 expression. J Agric Food Chem.

[32] Zou J, Zhang S, Li P, Zheng X, Feng D (2018). Supplementation with curcumin inhibits intestinal cholesterol absorption and prevents atherosclerosis in high-fat diet-fed apolipoprotein E knockout mice. Nutr Res.

[33] Shakeri A, Cicero AFG, Panahi Y, Mohajeri M, Sahebkar A (2019). Curcumin: A naturally occurring autophagy modulator. J Cell Physiol.

[34] Carmeliet P (2005). Angiogenesis in life, disease and medicine. Nature.

[35] Yamagata K (2017). Docosahexaenoic acid regulates vascular endothelial cell function and prevents cardiovascular disease. Lipids Health Dis.

[36] Wang Z, Wang Y, Chen Y, Lv J (2016). The IL-24 gene protects human umbilical vein endothelial cells against H₂O₂-induced injury and may be useful as a treatment for cardiovascular disease. Int J Mol Med.

[37] Guo N, Chen F, Zhou J, Fang Y, Li H, Luo Y (2015). Curcumin attenuates rapamycin-induced cell injury of vascular endothelial cells. J Cardiovasc Pharmacol.

[38] Li J, Luo M, Xie N, Wang J, Chen L (2016). Curcumin protects endothelial cells against homocysteine induced injury through inhibiting inflammation. Am J Transl Res.

[39] Luo R, Zhao L, Li S, Chen P, Wang L, Yu H (2021). Curcumin alleviates palmitic acid-induced LOX-1 upregulation by suppressing endoplasmic reticulum stress in HUVECs. Biomed Res Int.

[40] Zingg JM, Hasan ST, Cowan D, Ricciarelli R, Azzi A, Meydani M (2012). Regulatory effects of curcumin on lipid accumulation in monocytes/macrophages. J Cell Biochem.

[41] Shin SK, Ha TY, McGregor RA, Choi MS (2011). Long-term curcumin administration protects against atherosclerosis via hepatic regulation of lipoprotein cholesterol metabolism. Mol Nutr Food Res.

[42] Mizushima N, Ohsumi Y, Yoshimori T (2002). Autophagosome formation inmammalian cells. Cell Struct Funct.

[43] Zhu W, Yuan Y, Liao G, Li L, Liu J, Chen Y (2018). Mesenchymal stem cells ameliorate hyperglycemia-induced endothelial injury through modulation of mitophagy. Cell Death Dis.

[44] Liao X, Sluimer JC, Wang Y, Subramanian M, Brown K, Pattison JS (2012). Macrophage autophagy plays a protective role in advanced atherosclerosis. Cell Metab.

[45] Razani B, Feng C, Coleman T, Emanuel R, Wen H, Hwang S (2012). Autophagy links inflammasomes to atherosclerotic progression. Cell Metab.

[46] Choi SE, Lee SM, Lee YJ, Li LJ, Lee SJ, Lee JH (2009). Protective role of autophagy in palmitate-induced INS-1 beta-cell death. Endocrinology.

[47] Wu H, Wang MC, Bohmann D (2009). JNK protects Drosophila from oxidative stress by trancriptionally activating autophagy. Mech Dev.

[48] Han J, Pan XY, Xu Y, Xiao Y, An Y, Tie L (2012). Curcumin induces autophagy to protect vascular endothelial cell survival from oxidative stress damage. Autophagy.

[49] Wang G, Zhu Y, Li K, Liao B, Wang F, Shao L (2021). Curcumin-mediated photodynamic therapy inhibits the phenotypic transformation, migration, and foaming of oxidized low-density lipoprotein-treated vascular smooth muscle cells by promoting autophagy. J Cardiovasc Pharmacol.

[50] Inoki K, Kim J, Guan KL (2012). AMPK and mTOR in cellular energy homeostasis and drug targets. Annu Rev Pharmacol Toxicol.

[51] Kim J, Kundu M, Viollet B, Guan KL (2011). AMPK and mTOR regulate autophagy through direct phosphorylation of Ulk1. Nat Cell Biol.

[52] Mizushima N, Levine B, Cuervo AM, Klionsky DJ (2008). Autophagy fights disease through cellular self-digestion. Nature.

[53] Chen WR, Yang JQ, Liu F, Shen XQ, Zhou YJ (2020). Melatonin attenuates vascular calcification by activating autophagy via an AMPK/mTOR/ULK1 signaling pathway. Exp Cell Res.

[54] Li Y, Chen C, Yao F, Su Q, Liu D, Xue R (2014). AMPK inhibits cardiac hypertrophy by promoting autophagy via mTORC1. Arch Biochem Biophys.

[55] Guo S, Long M, Li X, Zhu S, Zhang M, Yang Z ( 2016 ). Curcumin activates autophagy and attenuates oxidative damage in EA hy926 cells via the Akt/mTOR pathway. Mol Med Rep.

